# Twenty-fifth Annual Report of the Managers of the New-York Asylum for Lying-in Women

**Published:** 1848-10

**Authors:** 


*Twenty-fifth Annual Report of the Managers of the New-York Asylum for Lying-in Women. Presented March* 22, 1848.

The following extracts from the above report, exhibit the operations of the charity during the year:—

"It appears that ninety-five patients have been admitted during the year, two of whom did not prove proper objects of the charity. The majority of the cases have been natural—five have been deviations. The results have been favorable, although some cases assumed a threatening character; their unpleasant symptoms have yielded to treatment, and we have gratification of reporting a successful termination.

"The Board, with you, have to lament the loss of one of their active and useful attending physicians, in the death of Dr. Washington. He was a vigilant officer of this institution, seeking by his medical efforts to render it every aid, by promptly attending to its calls. We trust and hope that he has received the reward of well doing.

"The place of Dr. Washington has been supplied by the appointment of Dr. Thomas F. Cock, who has the unanimous approbation of the Board.

"Three hundred and forty-two applications have been answered in the out door department during the past year, and it is gratifying to state that, notwithstanding the neglect, exposure, and improper nourishment to which many of the applicants have been subjected, so small a proportion of deaths have been reported by the district physicians.

"For this favorable report the recipients of this part of the charity are much indebted to the promptness and skill of the gentlemen composing the district corps of medical officers. Our confidence still continues in the arrangement of our different departments, and we submit this report with increased expectations of its future usefulness."

It appears from another portion of the report, that of *one thousand nine hundred and eighty-two patients* that have been received into the institution, from its commencement, there have been *twenty-nine* deaths.

The charity appears to be sustained by lady subscribers, the names of whom are appended to the report. We wish the report might be in the hands of every philanthropic lady of this, and other towns, so that they might be induced thereby to go and do likewise.

